# Longitudinal live imaging of retinal α-synuclein::GFP deposits in a transgenic mouse model of Parkinson’s Disease/Dementia with Lewy Bodies

**DOI:** 10.1038/srep29523

**Published:** 2016-07-08

**Authors:** Diana L. Price, Edward Rockenstein, Michael Mante, Anthony Adame, Cassia Overk, Brian Spencer, Karen X. Duong-Polk, Douglas Bonhaus, James Lindsey, Eliezer Masliah

**Affiliations:** 1 Neuropore Therapies, Inc., CA 92121, San Diego, USA; 2 Department of Neurosciences, University of California, La Jolla, CA 92093, San Diego, USA; 3 Department of Ophthalmology, University of California, La Jolla, CA 92093, San Diego, USA; 4 Department of Pathology, University of California, La Jolla, CA 92093, San Diego, USA

## Abstract

Abnormal α-synuclein (α-syn) accumulation in the CNS may underlie neuronal cell and synaptic dysfunction leading to motor and cognitive deficits in synucleinopathies including Parkinson’s disease (PD) and Dementia with Lewy Bodies (DLB). Multiple groups demonstrated α-syn accumulation in CNS accessory structures, including the eyes and olfactory terminals, as well as in peripheral organs of Parkinsonian patients. Retinal imaging studies of mice overexpressing fused α-syn::GFP were conducted to evaluate the presence and progression of retinal pathology in a PD/DLB transgenic mouse model. Bright-field image retinal maps and fluorescent images were acquired at 1-month intervals for 3 months. Retinal imaging revealed the accumulation of GFP-tagged α-syn in retinal ganglion cell layer and in the edges of arterial blood vessels in the transgenic mice. Double labeling studies confirmed that the α-syn::GFP-positive cells were retinal ganglion cells containing α-syn. Accumulation of α-syn persisted in the same cells and increased with age. Accumulation of α-syn::GFP was reduced by immunization with single chain antibodies against α-syn. In conclusion, longitudinal live imaging of the retina in the PDGF-α-syn::GFP mice might represent a useful, non-invasive tool to monitor the fate of α-syn accumulation in the CNS and to evaluate the therapeutic effects of compounds targeting α-syn.

Abnormal accumulation of α-synuclein (α-syn) is hypothesized to underlie the dopaminergic and non-dopaminergic neuronal cell death and synaptic dysfunction leading to motor and cognitive symptoms in Parkinson’s disease (PD), PD dementia (PDD) and Dementia with Lewy Bodies (DLB)^1^,^2^,^3^,^4^. Jointly, this heterogeneous group of disorders is referred to as Lewy body disease (LBD)^5^. Although no model reproduces all disease relevant features, transgenic (tg) mouse models overexpressing α-syn have proved useful in characterizing specific behavioral, neuropathological, and biochemical consequences of α-syn aggregation (comprehensively overviewed^6^). An ongoing effort in the field has been to find disease-relevant features in these transgenic mouse models with translational value for clinical trials in patients. Recent studies in the mThy1-α-syn transgenic (tg) (line 61) have revealed potentially clinically translatable alterations in bowel motility^7^, olfactory function^8^, hemodynamics^9^, and sleep disorders^10^, and these features parallel some the early and persistent symptoms in patients.

In the search for translatable biomarkers, recent studies have investigated the patterns of α-syn accumulation in accessory structures of the CNS such as the eyes^11^,^12^ and olfactory terminals^13^ and in peripheral organs such as the gut^14^,^15^, skin^16^, heart^17^, and salivary glands^18^. Among them, ophthalmologic alterations might be of interest because of its close proximity and connections between the eyes and the CNS. Varied degrees of changes in retinal structure and/or functional visual impairment have been observed in Parkinsonian patients and patients with other neurodegenerative diseases^19^,^20^,^21^,^22^,^23^,^24^,^25^. Furthermore, recent studies have shown the presence of α-syn deposits in the retina in PD patients^11^,^12^.

In this context, we evaluated a transgenic mouse model of PD/DLB for the presence and quality of α-syn deposits in the retina in an effort to develop a non-invasive live imaging assay that will allow longitudinal studies of α-syn accumulation in the retina as a way to evaluate the effects of aging, as well as therapeutical agents. For this purpose, we conducted retinal imaging studies in mice overexpressing fused α-syn-eGFP (α-syn::GFP) under the PDGF-beta promoter (PDNG78 line)^26^. This transgenic mouse line was selected because it displays biochemical and neuropathological features consistent with DLB/PD and because we have previously shown that these mice are amenable for imaging in real time the fate of α-syn in the CNS *in vivio*^27^,^28^. Remarkably, retinal imaging at four separate time points over a three-month period revealed accumulation of GFP-tagged α-syn in retinal ganglion cells, and this accumulation of α-syn persisted in the same cells over time and increased with age, supporting the notion that this method and model could be a useful tool to monitor in a non-invasive manner α-syn accumulation in the CNS in a non-invasive manner.

## Materials and Methods

### Animals and immunization

UCSD is an Institutional Animal Care and Use Committee accredited institution and the Animal Subjects Committee approved the experimental protocol followed in all studies according to the Association for Assessment and Accreditation of Laboratory Animal Care International guidelines. All behavioral testing procedures were performed under UCSD IACUC Protocol S02221. For this study, transgenic mice expressing the fused α-syn::GFP under the PDGFβ promoter (PDNG78 line) were used^26^. This model was selected because as we have previously shown that the α-syn::GFP expression in the CNS is stable, localizes to the synapses, accumulates in the neuronal cells body and axons, and over time results in synaptic alterations and functional deficits^1^. Under this promoter α-syn::GFP expression is most abundant in the neocortex, limbic system and olfactory bulb and cortex. A total of 12 non-tg and 12 α-syn::GFP tg mice were utilized for the longitudinal imaging studies. At the beginning of the analysis mice were 2.5 months old, studies were performed for 3 months. An additional group of 24 mice (n = 12 non-tg and n = 12 tg mice; 12 months of age) were used in studies to evaluate the effect of a brain-penetrating α-syn antibody on the deposition of GFP-tagged α-syn in the retina. We have previously shown that the single chain antibody clone against aggregated α-syn (D5) containing a secretion signal (CD5) and a brain penetrating peptide (apoB fragment) produced peripherally following lentiviral vector injection reduces the accumulation of α-syn in CNS and related deficits^29^. In this context, non-tg and α-syn::GFP tg mice were injected IP (10 μL) with the LV-CD5-D5-apoB (40 MOI, 2 × 10 ^ 9) or LV-control (n = 6 per group). After 4 weeks, retinoscopy and image analysis were performed as described below.

### *In vivo* retinal imaging

**Figure 1:**
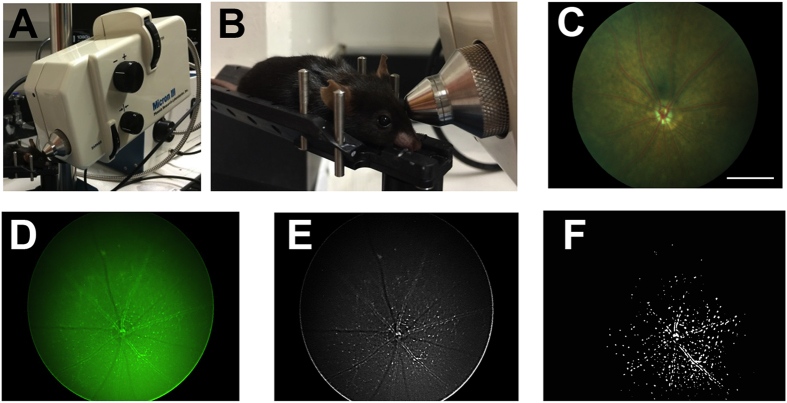
*In vivo* retinal imaging of α-syn::GFP transgenic and non-transgenic mice. (**A**) Xenon light source and a CCD-camera coupled microscope. (**B**) Placement of the mouse on the positioning table. (**C**) Mouse bright-field image retinal maps and (**D**) fluorescent retinal images in the same orientation for each eye. (**E**) Inverted grey scale image of retinal image. (**F**) Flattened image for analysis using a threshold set to maintain a dynamic range to enable comparison between retinae scans for various animals over time. Scale bar = 0.75 mm.

A Phoenix Micron III Retinal Imaging Microscope (Phoenix Research Labs, Pleasanton, CA) (Fig. 1A) was utilized for non-invasive bright-field and fluorescent retinal imaging studies in anesthetized α-syn::GFP transgenic and non-transgenic mice. The apparatus consists of a Xenon light source and a CCD-camera coupled microscope with a resolution of 4 μM in a field of view of 1.8 mm, which covered a 2.54 mm^2^ area (Fig. 1A). Just prior to imaging, mice were anesthetized with isoflurane (3%). The pupils of both eyes were then dilated using a solution of 1% atropine sulfate and 2.5% phenylephrine HCl solutions (Akorn Pharmaceuticals, Lake Forest, IL). Upon full pupillary dilation, animals were placed onto the positioning table (Fig. 1B), Gonak solution (2.5%) was applied to the eyes as a wetting and immersion media, and oriented for imaging was performed (Fig. 1B). Every effort was made to center the optic nerve in images; however, in a few cases images were slightly off-center. Mouse bright-field image retinal maps (normal scan mode) were acquired (Fig. 1C) for image registration and confirmation of eye clarity for fluorescent imaging. Fluorescent retinal images (Fig. 1D; progressive scans of 30) were then acquired in the same orientation for each eye. Consistent imaging angles were necessary to facilitate comparative analyses of images across *in vivo* imaging sessions. The gain and averaging image settings were kept consistent between subjects. The imaging session for both eyes of each animal typically took less than 5 minutes to acquire.

### Image analysis

Digital color fluorescent images of the retina obtained with the Micron III Retinal Microscope were first converted to gray scale images (Fig. 1E), inverted, and then flattened for analysis (Fig. 1F). The images were opened and batch processed by an observer blinded to any treatment information. For each image, a window with the original static image was kept adjacent to the threshold window and the threshold was only adjusted to match the original image by eye. Digital images were then analyzed with the Image J (NIH) program to ascertain the percent area of the retina that exhibited fluorescence above the threshold level and to count the total number of fluorescent particles. For this purpose a threshold was set to maintain a dynamic range that was comparable among the retinal scan for the various animals and time points analyzed.

### NRetina immunocytochemistry and laser confocal analysis

After the last *in vivo* scan, subjects were euthanized and whole eyes were dissected for each subject for immunocytochemical evaluations. As previously described^30^, the eyes were placed into oxygenated HEPES-buffered Ringer’s solution and the cornea, vitreous and lens were removed. The eye was cut open, and then the retina was detached from the eyecup with forceps. The retina was dissected into quarters. The retina was fixed for 60 min with 4% paraformaldehyde in phosphate buffer. After this, the tissue was immunostained as whole-mount for immunocytochemical analysis. For this purpose, the free floating retinal quarters were immunolabeled with antibodies against human α-syn (mouse monoclonal, SYN211 Abcam Ab80627, 1:1,100), Thy1/CD90 (mouse monoclonal Abcam Ab225, 1:500), phosphorylated neurofilaments (mouse monoclonal SMI312 Abcam Ab24574, 1:1000) and GFAP (rabbit polyclonal Abcam Ab7260, 1:1,000) followed by secondary antibodies tagged with biotin (1:100, Vector Laboratories, Inc., Burlingame, CA), Avidin D-HRP (1:200, ABC Elite, Vector) and detection with the Tyramide Signal Amplification™-Direct (Red) system (1:100, NEN Life Sciences, Boston, MA) mounted under glass coverslips with anti-fading media (Vector Laboratories). The labeled sections were then imaged with a laser scanning confocal microscope (LSCM) (MRC1024, BioRad). A subset of eyes were embedded in paraffin, serially sectioned at 10 μm and immunostained with antibodies against total (mouse and human) α-syn (SYN-1, BD, 1:500) followed by secondary antibodies tagged with biotin (1:100, Vector Laboratories, Inc., Burlingame, CA), Avidin D-HRP (1:200, ABC Elite, Vector) and detection with diaminobenzidine (DAB). These sections were analyzed with an Olympus BX50 digital microscope. Retinas from non-tg and α-syn tg mice treated with LV-control and LV-CD5-D5 were processed as described above as whole-mount free-floating and immunolabeled with antibodies against the V5 tag (red) to detect the presence of single chain antibody^31^ and the SYN-1 antibody to detect endogenous and tg α-syn (green). Following incubation with primary antibodies, free-floating retinas were incubated with tyramide red to detect the V5 antibody and with a monoclonal antibody against mouse α-syn tagged with FITC. Then retinas were mounted on to glass slides and covered with Vectashield (VectorLabs) and glass slides and images with the LSCM (MRC1024 BioRad). Then for each section 4 images, 1024 × 1024 pixels^2^, were obtained and analyzed with ImageJ to determine the average pixel intensity.

### Statistical analysis

All experiments were performed blind coded and all microscopic evaluations were imaged for 4 fields per slide. Values in the figures are expressed as means ± SEM. To determine the statistical significance, values were compared using Student’s T test, one-way ANOVA with repeated measures, or one-way ANOVA with *post hoc* Dunnett *post hoc* tests, as appropriate and indicated in the figure legends. The differences were considered to be significant if p-values were less than 0.05.

## Results

### *In vivo* imaging reveals α-syn deposits in the inner retina of the PDGFα-syn::eGFP transgenic mice

**Figure 2:**
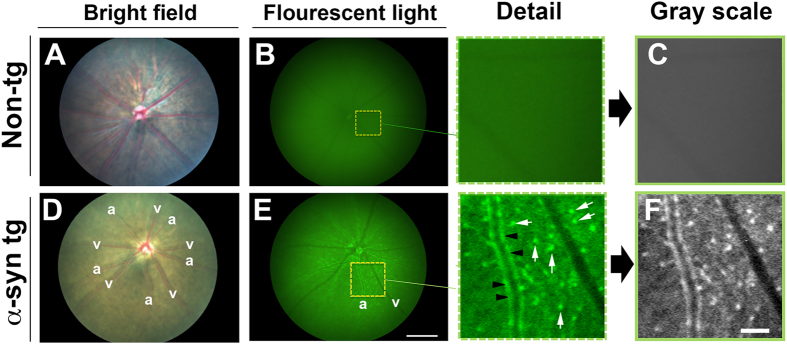
Visualization of α-syn deposits in the inner retina of the PDGFα-syn::eGFP transgenic mice. (**A**) Images of non-tg mice, which failed to show signal using bright-field imaging, (**B**) fluorescent light imaging, and (**C**) after gray scale image conversion. (**D**) Photomicrographs of α-syn::GFP transgenic optic discs and retinal vessels of normal characteristics. Arteries (a) and veins (v) were distinguished by their width. (**E**) Fluorescent and (**F**) Gray scale retinal imaging demonstrated the presence of bright abundant α-syn::GFP dot-like structures around blood vessels and in the interstitium (Detail; white arrows), likewise α-syn::GFP positive signal was detected in the wall of vessels (Detail; black arrowheads) that appear to be arteries. There was little or no detectable α-syn::GFP around veins. Scale bar in E = 0.75 mm; scale bar in F = 70 μm.

By bright-field imaging of the retinal fundus, the non-tg (Fig. 2A) and α-syn::GFP tg mice (Fig. 2D) showed optic discs and retinal vessels of normal characteristics. Arteries (a) and veins (v) were distinguished by their width; arteries were thinner compared to veins. In the fluorescent mode of the retinoscope, the non-tg mice did not show any signal (Fig. 2B,C); however, in the α-syn::GFP tg mice (Fig. 2E,F) retinal imaging demonstrated the presence of bright abundant α-syn::GFP dot-like structures around blood vessels and in the interstitium (white arrows), likewise α-syn::GFP positive signal was detected in the wall of vessels (black arrowheads) that appear to be arteries. There was little or no detectable α-syn::GFP around veins (Fig. 2E,F).

**Figure 3:**
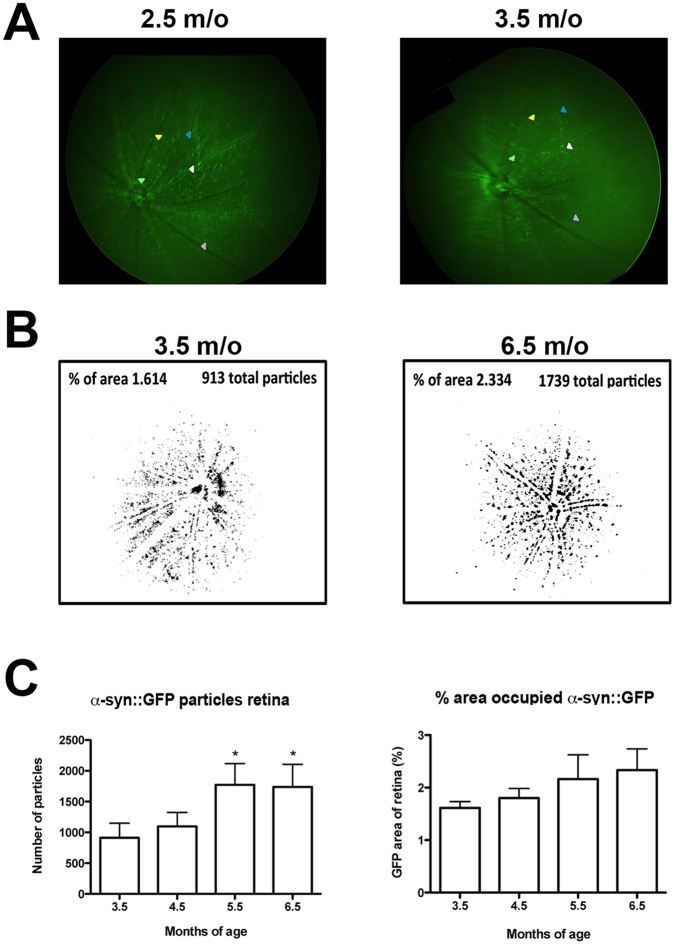
α-Syn::GFP increases with age in tg mice. (**A**) Fluorescent images of persistent α-syn::GFP dot-like structures in same mouse visualized 1 month apart. The coinciding features are marked with matched arrowhead colors. (**B**) Representative images from one subject per group along with the group means for the percentage of area with α-syn::GFP, as well as total number of particles present increased over 3 months. (**C**) The number of α-syn::GFP particles remained similar between 3.5 and 4.5 and then significantly increased by 5.5 months of age and remained elevated at 6.5 months of age compared to 3.5 months of age. There was a non-significant trend toward an increase in the area occupied by α-syn::GFP with age. Data were analyzed using repeated measures one-way ANOVA with Dunnett’s post hoc comparisons (criteria for significance was set at p < 0.05). *p < 0.05. N = 12.

Three additional retinoscopy imaging sessions were conducted at one month intervals following the first baseline imaging session to evaluate the persistence of specific α-syn::GFP dot-like structures in the retina in the same mice over time with a consistent imaging angle. Longitudinal imaging studies of the mice demonstrated the presence of persistent α-syn::GFP dot-like structures in same mouse; the coinciding features are marked with matched arrowhead colors (Fig. 3A). These longitudinal evaluations illustrated the necessity of a consistent imaging angle for repeat imaging of the mouse retina.

Finally, we wanted to determine the effects of time upon the α-syn::GFP signal when performing longitudinal *in vivo* retinal evaluations. For this purpose within-mouse comparisons of images α-syn::GFP tg mice were collected over a three-month period (starting at 3.5 m/o). The image analysis package ImageJ (NIH) was utilized to determine the total number of particles present, as well as the percentage of area with α-syn::GFP. Representative images from one subject per group are shown together with the group means for these measures (Fig. 3B). For particle counts between 3.5 and 4.5 months of age there was no significant difference. However, at 5.5 months of age there was a significant increase that continued through 6.5 months compared to 3.5 months of age using repeated measures one-way ANOVA with Dunnett’s post hoc analysis (Fig. 3C). For the percent area of retina with α-syn::GFP there was a progressive non-significant increase from 3.5 to 6.5 months of age (Fig. 3C) by repeated measures one-way ANOVA with Dunnett’s post hoc analysis. This study revealed age-dependent increases in α-syn::GFP in tg mice (Fig. 3B,C). Together, these studies utilizing the Micron III retinoscopy system indicate that in tg mice dot-like structure are detected in the retina and that these α-syn::GFP features are persistent and increase over time.

### Immunocytochemical analysis localizes α-syn::GFP to retinal ganglion cells

**Figure 4:**
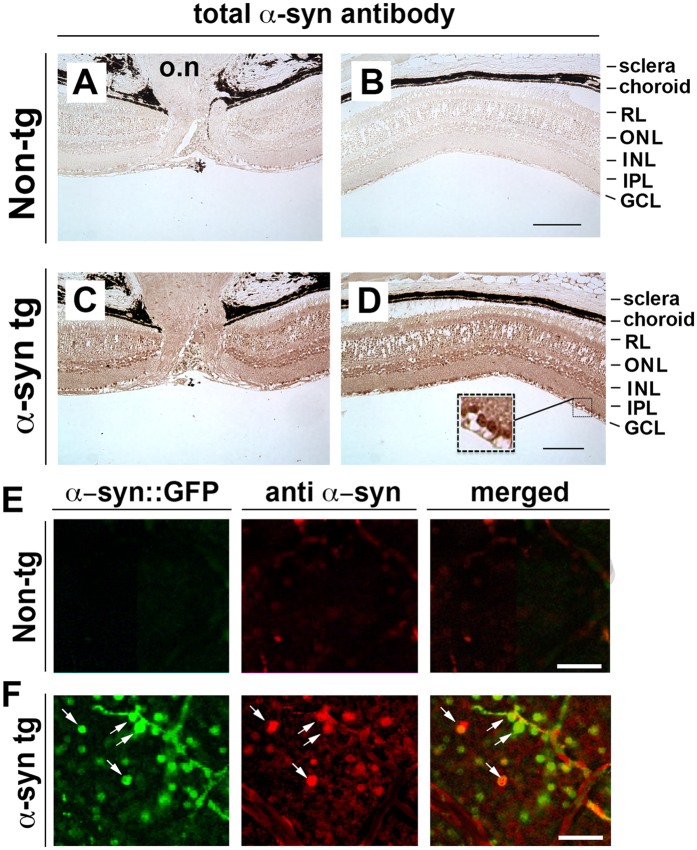
α-Syn::GFP dot-like structures detected in the retinoscopy are retinal ganglion cells. (**A**,**B**) Photomicrographs of paraffin sections immunostained with an antibody against α-syn in the non-tg mice revealed little to no immunoreactivity, while (**C**,**D**) in the α-syn::GFP tg mice α-syn immunostaining was present in the various layers of the retina with the strongest immunoreactivity detected in the INL and in the GCL. In the GCL, strong immunostaining was associated with the cell bodies and nucleus (inset). (**E**) Laser confocal microscopy analysis of retina whole mounts from non-tg mice revealed no detectable immunoreactivity for either α-syn::GFP or α-syn; while (**F**) the tg mice the α-syn::GFP-positive cells were co-labeled with the same antibody against α-syn. RL, receptor layer; ONL, outer nuclear layer; INL, inner nuclear layer; IPL, inner plexiform layer GCL, ganglion cell layer. Bright-field scale bar = 70 μm; Fluorescent scale bar = 40 μm.

**Figure 5:**
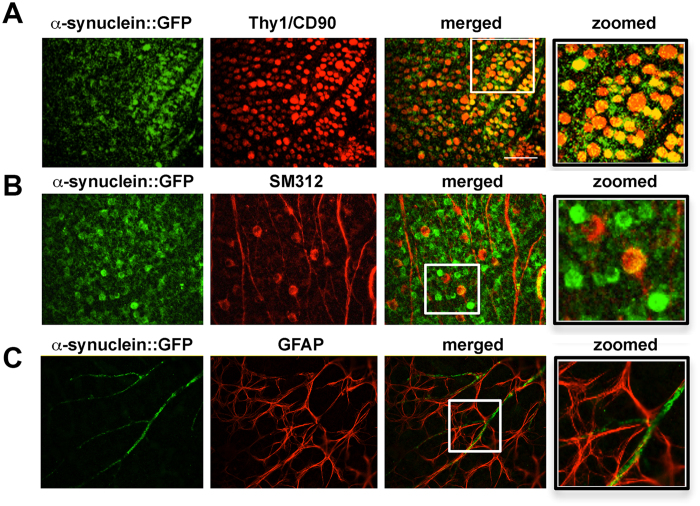
Co-labeling and confocal microscope imaging showing α-syn::GFP immunoreactivity in retinal ganglion cells. (**A**) Whole retina mount of α-Syn::GFP positive cells (green) were co-labeled with the retinal ganglion cell (RGC) markers thy-1/CD90 (red) and (**B**) neurofilaments (red). (**C**) α-Syn::GFP positive processes (green) were not co-labeled with the astroglial cell marker GFAP (red). Scale bar = 40 μm.

To determine the cellular identity of the α-syn::GFP dot-like structures in the retina of the tg mice and also to confirm that the fluorescent signal captured in the *in vivo* imaging was due to GFP-tagged α-syn and not free GFP or an artifact, immunocytochemical studies with antibodies against α-syn, Thy1/CD90, neurofilaments (NFs) and GFAP were performed. By bright-field digital microscopy using paraffin sections immunostained with an antibody against α-syn in the non-tg mice, mild immunoreactivity was detected (Fig. 4A,B), in contrast in the α-syn::GFP tg mice there was α-syn immunostaining in the various layers of the retina with the strongest immunoreactivity detected in the inner nuclear layer (INL) and in the ganglion cell layer (GCL) (Fig. 4C,D). In the GCL, strong immunostaining was associated with the cell bodies and nucleus (Fig. 4D, inset). Laser confocal microscopy analysis of retina whole mounts showed that compared to non-tg mice where no labeling was detected (Fig. 4E); in the tg mice the α-syn::GFP positive cells were co-labeled with the same antibody against α-syn (Fig. 4F) that was used for the paraffin sections. Additional immunolabeling and laser confocal microscopy analysis of retina whole mounts showed that in the tg mice, the α-syn::GFP positive cells were co-labeled with the retinal ganglion cell (RGC) markers thy-1/CD90 (Fig. 5A) and neurofilaments (Fig. 5B) but not with the astroglial cell marker GFAP (Fig. 5C). Taken together, these studies suggest that the α-syn::GFP dot-like structures detected in the retinoscopy are RGCs.

### Immunotherapy with brain penetrating single chain antibodies against α-syn reduces α-syn accumulation in the retina of transgenic mice

**Figure 6:**
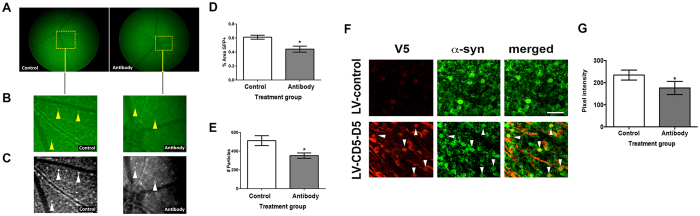
Single chain antibodies directed against oligomeric α-syn significantly reduced α-syn::GFP burden in the retina. (**A**) Florescent images of the whole retina from tg mice treated with the LV-control displayed abundant α-syn::GFP dot-like structures in the retina compared to the tg mice treated with LV-CD5-D5-apoB (antibody). (**B**) Higher magnification of retina area marked with a dotted square in panel **A**. Arrowheads indicate α-syn::GFP-positive structures. (**C**) Black and white enhanced images of detail in panel **A**. (**D**) LV-CD5-D5-apoB significantly reduced the percent area of the retina occupied by GFP particles compared to LV-control injection. (**E**) LV-CD5-D5-apoB significantly reduced the number of GFP particles that occupied the retina compared to LV-control injection. (**F**) Immunocytochemical analysis in whole mount retina from the tg mice showed reduced levels of α-syn immunoreactivity in the GCL in mice treated with the LV-CD5-D5-apoB compared to LV-control. The single chain antibody colocalized with α-syn antibody showing that the single chain antibody labeled with an antibody against V5 colocalized to α-syn containing retinal ganglion cells. Arrowheads identify the same structures in each panel for which the single-chain antibody colocalizes with α-syn. (**G**) Image analysis for the levels of α-syn-immunoreactivity expressed as pixel intensity comparing the LV-control with LV-CD5-D5-apoB treated animals. Statistical analysis was performed using Student’s T with differences considered to be significant if p-values were less than 0.05. *p-value < 0.05 compared to LV-control injection. Scale bar = 40 μm. N = 6 per group.

We have previously shown that active and passive immunization might be a potential treatment modality for synucleinopathies^32^. Among the various possible approaches, we have recently shown that single chain antibodies directed against oligomeric α-syn (clone CD5-D5) expressed peripherally with lentiviral vectors (LV-CD5-D5-apoB) penetrates into the CNS and reduces the accumulation of α-syn and related deficits in tg mice^29^. In this case we enhanced the brain penetrating capacity of the single chain antibodies by adding a 38aa domain of apolipoprotein B (apoB)^29^. In this context, and as a proof of concept of the utility and specificity of the retinoscopy approach to monitor therapeutical effects, α-syn::GFP tg mice received an IP injection with either LV-control or LV-CD5-D5-apoB and then evaluated 1 month later. As expected, the tg mice treated with the LV-control displayed abundant α-syn::GFP dot-like structures in the retina one month after the injection (Fig. 6A–C, control), which occupied 0.6% of the area (Fig. 6D) and with an average of 500 particles per unit area (Fig. 6E). In contrast mice treated with LV-CD5-D5-apoB showed a reduction (Fig. 6A–C, Antibody) with an average 0.4% of the area of the retina occupied by GFP particles (Fig. 6D) and an average of 380 particles per unit area (Fig. 6E). Similarly, immunocytochemical analysis of whole mount sections from the tg mice showed reduced levels of α-syn immunoreactivity in the GCL in mice treated with the LV-CD5-D5-apoB compared to LV-control (Fig. 6F). Levels of α-syn immunoreactivity were decreased in the antibody-treated group compared to the control-treated group (Fig. 6G). Taken together, these results suggest the retinal imaging in the α-syn::GFP mouse follows α-syn aggregation in the retinal ganglion cells and this could be used to monitor the effect of treatments such as immunotherapy.

## Discussion

The findings of the present study supports the possibility that live real-time imaging of the retina of the α-syn::GFP tg mice might be a novel non-invasive approach to monitor α-syn deposition and therapeutic effects in the CNS. We showed using retinal imaging that the α-syn::GFP tg mice display α-syn deposits that were persistent and increased over time. The α-syn::GFP deposits co-localized with markers for the RGC, and immunotherapy with a brain penetrating single chain antibody against α-syn oligomers decreased the α-syn::GFP signal in the retina of tg mice. The localization of α-syn::GFP signal to RGC is consistent with previous immunocytochemical studies that have found that transgenic mice overexpressing α-syn under various promoters display the presence of immuno-positive deposits in the optic nerve and accumulation of immunoreactivity in specific retinal cells^33^. Similarly, studies in α-syn tg drosophila have shown considerable ophthalmologic alterations with retinal degeneration^34^. Moreover, mis-expression of Tau and α-syn enhanced a rough eye phenotype and loss of dopaminergic neurons in drosophila models of tauopathy and synucleinopathy. The authors of those studies concluded that interactions between α-syn and Tau disrupt the organization of the cytoskeleton causing axonal transport defects and aberrant synaptic organization that contribute to neuronal dysfunction^35^.

We have previously shown that in this transgenic mouse model, expression of α-syn::GFP under the control of the PDGFβ promoter results in the accumulation of α-syn in the inner layers of the neocortex and limbic system and to a lesser extent in the striato-nigral system^26^. In fact, recent studies have demonstrated that such deposits in the neocortex can be imaged in real time by two-photon microscopy^27^,^28^. In addition, these mice display hippocampal synaptic dysfunction, as well as behavioral impairments^36^. Together these findings suggest that the α-syn::GFP tg model might mimic some of the non-motor aspects of LBD^6^,^37^. In addition to the dopaminergic alterations, patients with LBD display α-syn pathology that could involve non-nigral brainstem nuclei, sympathetic, parasympathetic, enteric and pelvic plexuses, cardiac systems, submandibular gland, adrenal medulla, skin as well as the retina^38^.

For example, a recent study found that patients with either PD or DLB display α-syn aggregates in the nerve fibers in the inner retinal surface suggestive of either retinopetal/centrifugal fibers or of ganglion cell axons^11^. In agreement with this report a more recent study found α-syn deposits in the inner retina^12^. These α-syn deposits were localized to discrete neurons within the inner retina^12^. Likewise another study reported pale α-syn positive inclusions in the outer plexiform layer of the retina in a patient with DLB^39^. Similarly, α-syn and ubiquitin inclusions were found in the inner nuclear layer in aged individuals. The proportion of patients displaying such α-syn and/or ubiquitin intracytoplasmic inclusions was significantly higher with advanced age^40^. These studies concluded that α-syn deposition in the retina might participate in the visual impairment in LBD. In contrast, a more recent study^41^ reported that eyes of patients with PD lacked definite Lewy bodies or Lewy neurites; however, patchy cytoplasmic α-syn aggregates were detected. Likewise, studies of the aging retina have shown increased α-syn and ubiquitin inclusions in the inner nuclear layer. The proportion of patients displaying such α-syn and/or ubiquitin intracytoplasmic inclusions was significantly higher with aging but distinct to what is found in PD. This suggests that although there is a slight increase in the levels of α-syn during normal ageing^40^, this increase is compensated by clearance mechanisms, while in PD it is possible that such mechanisms fail and α-syn accumulates intracellular, leading to degeneration. Using a phosphorylation-independent antibody, tau aggregates were observed within the cytoplasm of several photoreceptor cells, and there was a positive correlation between age and the number of tau-positive ganglionic cells^40^. In addition to α-syn deposits in the RGC, we also detected α-syn-GFP signal along the wall of the blood vessels and more prominently in the arteries. These findings are consistent with a previous study which reported α-syn deposition in human cerebral blood vessels^42^. The study by Tamo *et al*. showed that α-syn could be expressed in vascular endothelial and smooth muscle cells. Other amyloidalgenic proteins such as Aβ and tau^43^ have been shown to distribute around blood vessels. While extracellular α-syn can be cleared via the perivascular interstitial space, further investigation is needed to understand the exact mechanism for and significance of α-syn deposition in blood vessels.

Under physiological conditions, α- and β-synucleins are present at low levels in the inner plexiform layer (IPL), whereas gamma-synuclein is in the nerve fiber layer^33^. In transgenic mice overexpressing α-syn, a different pattern of localization depending on the promoter used for the expression was observed^44^. Gamma-synuclein is highly expressed in RGCs in the human retina and colocalized with Brn-3a, and axons of RGCs were immunopositive for gamma-synuclein in the nerve fiber layer (NFL)^45^. In agreement with these studies of protein localization in the retina, gene-profiling studies have shown that α-syn mRNA and protein were expressed by both retinal pigment epithelium (RPE) and RGC^46^. High levels of α-syn were also reported in the outer segments (OS) of photoreceptors and in their axon terminals in the outer plexiform layer (OPL) of the retina^46^. Moreover, α-syn and synaptophysin co-localized in retinal presynaptic terminals^46^.

Retinal imaging has been proposed as an alternative, non-invasive approach to monitor accumulation of protein aggregates in neurodegenerative disorders. For example, retinal imaging following curcumin administration has revealed plaque-like amyloid deposits in patients with AD and in APP/PS tg mouse models^24^,^47^. Our study proposes that similar approaches might be applicable to DLB and PD patients with the caveat that fluorescent imaging agents similar to curcumin that might be able to detect with sensitivity and specificity α-syn in the retina are needed. In summary, studies support the use of the PDNG78 transgenic mouse as a model system to evaluate α-syn -associated retinal pathology as a surrogate marker for CNS neuropathology and to evaluate therapeutic evaluations.

## Additional Information

**How to cite this article**: Price, D. L. *et al*. Longitudinal live imaging of retinal α-synuclein::GFP deposits in a transgenic mouse model of Parkinson’s Disease/Dementia with Lewy Bodies. *Sci. Rep.* **6**, 29523; doi: 10.1038/srep29523 (2016).
